# Regional VRE surveillance using routine centralised, multicentre whole genome sequencing

**DOI:** 10.1371/journal.pone.0334734

**Published:** 2026-06-25

**Authors:** Djamayl L. H. H. Engelen, Jean-Luc Murk, Veronica A. T. C. Weterings, Heike Schmitt, Bram M. W. Diederen, Andreas L. E. van Arkel, Jaco J. Verweij, Wouter van den Bijllaardt, Jeroen H. T. Tjhie, Joep J. J. M. Stohr

**Affiliations:** 1 Microvida, Laboratory of Medical Microbiology, Amphia Hospital, Breda, The Netherlands; 2 Microvida, Laboratory of Medical Microbiology and Immunology, Elisabeth-TweeSteden Hospital, Tilburg, The Netherlands; 3 Microvida, Laboratory of Medical Microbiology, Bravis Hospital, Roosendaal, The Netherlands; 4 Microvida, Laboratory of Medical Microbiology, Admiraal de Ruyter Hospital, Goes, The Netherlands; 5 Microvida, Laboratory of Medical Microbiology, ZorgSaam Hospital, Terneuzen, The Netherlands; 6 Department of infection prevention and control, Amphia Hospital, Breda, The Netherlands; 7 National Institute for Public Health and the Environment (RIVM) - Centre for Zoonoses and Environmental Microbiology, Bilthoven, The Netherlands; 8 Department of infection prevention and control, Bravis Hospital, Roosendaal, The Netherlands; 9 Department of infection prevention and control, Admiraal de Ruyter Hospital, Goes, The Netherlands; 10 Department of infection prevention and control, Elisabeth-TweeSteden Hospital, Tilburg, The Netherlands; Charite Universitatsmedizin Berlin, GERMANY

## Abstract

**Objective:**

Whole genome sequencing (WGS) is increasingly used to support infection prevention and control. However, its application for routine regional surveillance of vancomycin-resistant Enterococcus faecium (VREfm) across multiple healthcare facilities is still evolving.

This study describes the implementation of routine centralised multicenter WGS surveillance of VREfm and explores its utility for detecting clusters and investigating transmission within and between healthcare institutions in a regional healthcare network.

**Methods:**

VREfm isolates identified from patient samples (clinical and screening) in five different hospitals in the Netherlands during a one-year period were whole genome sequenced and typed using core genome multilocus sequence typing (cgMLST). Sequence data were correlated with admission data of the year prior to the first positive VREfm culture of the respective patients. By combining epidemiological and sequence data within and between hospital transmission events were identified.

**Results:**

57 VREfm isolates were detected during the study period. 38/57 (66.7%) isolates clustered with at least one other isolate. Based on our definitions, intrahospital transmission could be demonstrated in 37 of 38 cases and interhospital transmission in 3 of 38 cases. Using this approach, a multicenter outbreak and two local outbreaks were detected.

**Conclusions:**

Routine whole genome sequencing of VREfm is a powerful tool for detecting, tracing and delineating outbreaks. When integrated into a centralised surveillance system spanning collaborative healthcare networks, it becomes an essential tool for unraveling the complex transmission dynamics of VRE within and between hospitals, ultimately strengthening infection control strategies.

## Introduction

Enterococci are one of the top three micro-organisms reported in hospital-acquired infections [1]. Vancomycin-resistant enterococci (VRE) are associated with increased morbidity and mortality compared to infections with vancomycin-susceptible enterococci [2–5]. In the Netherlands, the overall prevalence of vancomycin-resistant Enterococcus faecium (VREfm) colonization is unknown but studies performed in high-risk hospital wards report a prevalence of 1–8.6% [6,7]. Furthermore, the prevalence of VREfm isolates in blood cultures is low compared to the European Union/European Economic Area (EU/EEA), possibly reflecting low colonization prevalence [8,9]. Despite this, nosocomial outbreaks occur regularly with 137 VREfm outbreaks between 2012 and 2023 which accounts for 20–32% of all nosocomial outbreaks in the Netherlands. In 2021 and 2022 a total of nine outbreaks per year were reported [8]. In 2017, the last year of which aggregated molecular data of VREfm was available on a national level, 57,5% of VREfm carried the vanA gene, 41,5% carried the vanB gene and a small percentage carried both genes or the vanD gene. Additionally, the most prevalent ST-types were ST117 (found in 33 hospitals), ST203 (found in 26 hospitals), ST80 (found in 18 hospitals) and ST18 (found in 15 hospitals) [10]. To prevent nosocomial VRE transmission, guidelines suggest infection prevention and control measures, including contact tracings for high-risk contacts of VRE positive patients [11].

Whole genome sequencing (WGS) is increasingly used to guide infection prevention and control [12,13]. Recently, routine application of WGS for multi-drug-resistant organisms (MDROs) in intrahospital surveillance has shown that VREfm transmission can be detected earlier compared to retrospective sequencing triggered by epidemiological data [12]. However, its application for routine regional, interhospital surveillance of VREfm is still evolving. The present study describes the application of regional routine WGS of VREfm isolates across five hospitals in two provinces in the Netherlands, with the aim of exploring its utility for detecting transmission within and between hospitals.

## Methods

### Setting

This prospective cohort study was performed from January 1, 2022, to August 1, 2023, across five hospitals (Elisabeth-TweeSteden Hospital in Tilburg (hereafter called hospital C), Amphia Hospital in Breda (hereafter called hospital B), Bravis Hospital in Roosendaal (hereafter called hospital A), Admiraal de Ruyter Hospital in Goes (hereafter called hospital D), Zorgsaam Hospital in Terneuzen (hereafter called hospital E)), with a catchment area of 1.7 million people in the provinces North-Brabant and Zeeland in the Netherlands. The minimum and maximum distance between two hospitals is 30 km and 140 km respectively. The Elisabeth-Tweesteden Hospital is the reference centre for all complex trauma, neurological and neurosurgical patients in North Brabant.

In all five hospitals, patients are screened for VREfm when they meet criteria for screening based on national guidelines (Table 1) [11]. Cultures are taken as soon as possible upon admission and patients are isolated using contact precautions while results are pending (see S3 Table for the interval between admission and culturing for each patient). New unexpected VREfm carriage or infection can also be detected in routine clinical cultures. Whenever a new VREfm is detected in any of the hospitals (routine clinical cultures, screening cultures and contact tracing cultures), the isolate is sent to the Elisabeth-Tweesteden Hospital for sequencing and cluster analysis.

**Table 1:** Criteria for VREfm screening based on national guidelines.

Patients admitted more than 24 hours to a foreign hospital < 2 months before admittance
Patients admitted to a foreign hospital 2–12 months before admittance and underwent an invasive procedure in the foreign hospital
Patients transferred from a hospital with an active VREfm outbreak
Patients living in an asylum seekers’ centre

Patients colonised or infected by VREfm, are admitted to single patient rooms and nursed using contact precautions. The VREfm colonization status is communicated between infection control departments of the different hospitals when patients are transferred between different hospitals. During admission, VREfm positive patients are not re-tested in an attempt to delabel those patients. Instead, they are deemed VREfm positive during their admission. For patients admitted to the same room, contact tracings and cultures are performed when 1. VREfm is detected in samples of patients that were admitted to a shared patient room and were not nursed using contact precautions prior to VREfm detection and 2. when VREfm is detected in patients admitted to a single patient room after a new patient is already admitted to that room. In that case the latter patient is also included in contact tracings. Additionally, after cleaning and disinfection, VREfm environmental cultures of the room in which VREfm positive patients were admitted are performed. Only after negative cultures, the room is given free for the admission of new patients. When cultures show growth of VREfm, a new round of cleaning, disinfection and culturing is performed until negative cultures. In addition to contact tracings and environmental cultures, patients admitted to wards where VREfm transmission is active are cultured weekly (prevalence studies).

### Included patients

All patients (in- and outpatient) in whom a VREfm was detected in a sample sent for microbiological culture between January 1, 2022 and August 1, 2023, were included in the study. For each included patient, the admission history (admission and discharge dates, wards of admission and rooms of admission) of the past 365 days in all five hospitals was retrieved. Sample metadata (collection date, hospital where collection was performed, sampling site and patient location during sampling) from VREfm-positive samples were also registered.

### Detection of VREfm

When E. faecium were identified in samples using MALDI-TOF MS (Bruker Daltonics GmbH (Bremen, Germany)), susceptibility testing was performed using the BD Phoenix system (BD, Franklin Lakes, New jersey, US) or Vitek-2 (bioMérieux, Marcy-l’Étoile, France) depending on the hospital. For samples collected from sterile sites (e.g., blood cultures, cerebrospinal fluid), a confirmation test with a dose of 5 µg vancomycin disk was used for disk diffusion following EUCAST guidelines [14]. When reduced susceptibility to vancomycin was detected (MIC > 4 mg/L, zone<12 mm or fuzzy border for disk diffusion) according to EUCAST breakpoints, molecular confirmation of VREfm was performed using GeneXpert (a rapid cartridge based PCR platform) using the Xpert vanA/vanB kit (Cepheid, California, USA) following the manufacturer’s instructions or using an in-house real-time PCR detecting the vanA or vanB gene as previously described [15]. For VREfm screening cultures, 1 ml of eSwab (Copan Diagnostics, Corona, CA) medium or a small quantity of feces was incubated overnight in Tryptone Soya Broth (TSB) (Thermo Fisher Scientific Oxoid, Hampshire, UK) after which 10 µl was streaked on a chromogenic VRE agar (CHROMagar VRE (Saint-Denis, FR)). This agar was then evaluated for VREfm-suspected colonies for 2 consecutive days. VREfm was confirmed as described.

### Molecular typing and cluster analysis

Isolates were suspended in 800 µL TE (Tris-EDTA) buffer until a milky-white suspension was obtained. DNA extraction was performed using the QIAsymphony DSP Virus/Pathogen Midi Kit (QIAGEN, Hilden, Germany) with the “Complex400_V4_DSP” protocol on the QIAsymphony sample processing system, using an elution volume of 110 µL. DNA concentration was measured using a Qubit 4 Fluorometer with the Qubit 1X dsDNA HS Assay Kit (Thermo Fisher Scientific, USA) and normalized to 0.2 ng/µL using nuclease-free water (QIAGEN). DNA tagmentation, library preparation, and cartridge loading were performed using Nextera XT chemistry and the MiSeq Reagent Kit v2 Micro according to the manufacturer’s instructions (Illumina, San Diego, CA, USA).

Whole genome sequencing was performed for the first VREfm isolate of each included patient on an Illumina MiSeq platform using the MiSeq Reagent Micro Kit v2. Trimming and assembly (based on de Bruijn graph) was performed using CLC Genomic Workbench (QIAGEN, Venlo, Netherlands). More information regarding our sequencing workflow can be found in the supplementary information (S1 and S2 Tables). The following quality control criteria were applied: coverage ≥ 30x, number of contigs ≤300, N50 ≥ 15,000 bp and maximum contig length ≥50,000 bp. Species-specific core-genome multilocus sequence typing (cgMLST) was performed for all sequenced isolates using Ridom SeqSphere+ (Ridom, Münster, Germany) with a validated Enterococcus faecium cgMLST scheme [16]. Minimum accepted percentage of cgMLST targets was 98%. Within SeqSphere + , pairwise genetic distances were calculated based on allelic differences. Clonality was defined as ≤20 allelic differences as recommended by the scheme developers, and isolates meeting this threshold were assigned identical local cluster types [16]. Time from detection of VREfm to the results from cluster analysis took around 2–3 days, and reports were sent directly to infection control teams. Minimum spanning trees (MST) were constructed from cgMLST allelic profiles using the MSTreeV2 algorithm implemented in GrapeTree (version 2.2) [17]. The resulting tree was exported in Newick format and visualized using the web version of SPREAD [18].

### Data analysis

Possible transmission events were investigated using admission and sequence data for each new VREfm-positive patient. For each cluster type, the epidemiological relationship between the patients was evaluated with a look-back period of 1 year before the first positive VREfm culture. Intra- and interhospital transmission was investigated for clonal isolates and definitions are given in Table 2.

**Table 2:** Definitions of transmission events.

Definition	Description
Probable intrahospital transmission	Patients colonised/infected with clonal VREfm isolates admitted to same hospital + same ward within one month of each other.
Probable interhospital transmission	Patients colonised/infected with clonal VREfm isolates admitted to two different hospitals with probable intrahospital transmission in both hospitals.

### Ethics

The study was evaluated by the regional medical ethics committee METC Brabant (NW2023−35) which waived the need for patient consent. Data was anonymized before analysis.

## Results

In the study period, 57 patients had a newly detected VREfm (S3 Table). In one patient, VREfm was isolated in a scrotal wound sample, in one in a blood culture, in 54 in rectal/perineal swabs, and in one in a stool sample. Additionally, in two patients VREfm was also isolated in a urine sample. None of the identified VREfm-positive patients were previously known to be infected or colonised with VREfm. 43 patients were admitted to only one hospital in the year before their first positive VREfm culture, four patients were admitted to two different hospitals, and 10 were never admitted to a hospital in the year prior to their first positive culture (S3 Table). The latter patients were sampled outside the hospital (n = 4 in a rehabilitation center and n = 1 by a primary care physician), in an outpatient clinic (n = 2), or were screened upon admission (and placed in contact isolation while awaiting culture results) because they were previously admitted to a hospital in another country but were not admitted to any of the included hospitals in the year prior to the screening (n = 3).

**Fig 1:**
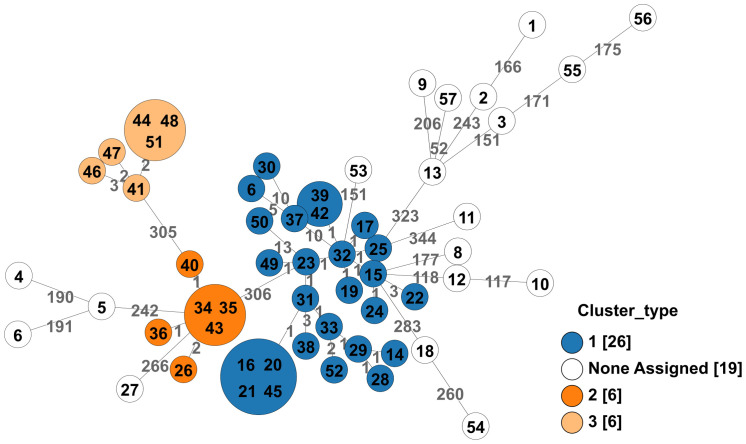
Minimum Spanning Tree (MST) of all VREfm isolates.

In 52 isolates, a vanA gene was detected and in 5 isolates a vanB gene. For 51 isolates a sequence type (ST) could be assigned amounting to 7 different STs. 38 of 57 VREfm isolates (66.7% of all patients, 84.4% of admitted patients) were clonally related to at least one other isolate in the study. Three different clusters of clonally related isolates were identified: cluster C1 (n = 26 isolates); cluster C2 (n = 6 isolates), and cluster C3 (n = 6 isolates). Isolate-pairs of the same cluster had a median genetic distance of one allele (range 0–16 alleles difference). Isolate-pairs not belonging to the same cluster had a median genetic distance of 367 alleles (range 66–541 alleles difference) (Fig 1). C1 belonged to ST-type 117 and carried the vanA gene. C2 belonged to ST-type 80 and also carried the vanA gene. C3 did not belong to a known ST-type and also carried the vanA gene.

**Fig 2:**
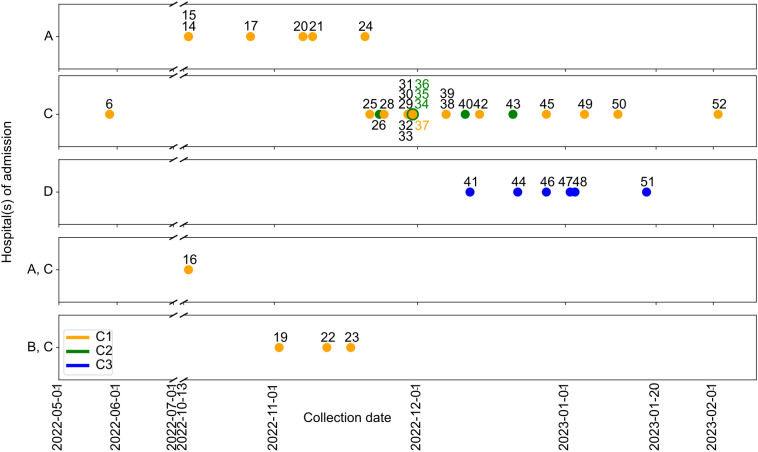
Admission data of VREfm positive patients of which their isolate clustered with at least another VREfm isolate. Numbers correspond to the respective VREfm isolates in S3 Table. * Green numbers correspond to cluster C2. Yellow numbers to cluster C1.

**Fig 3:**
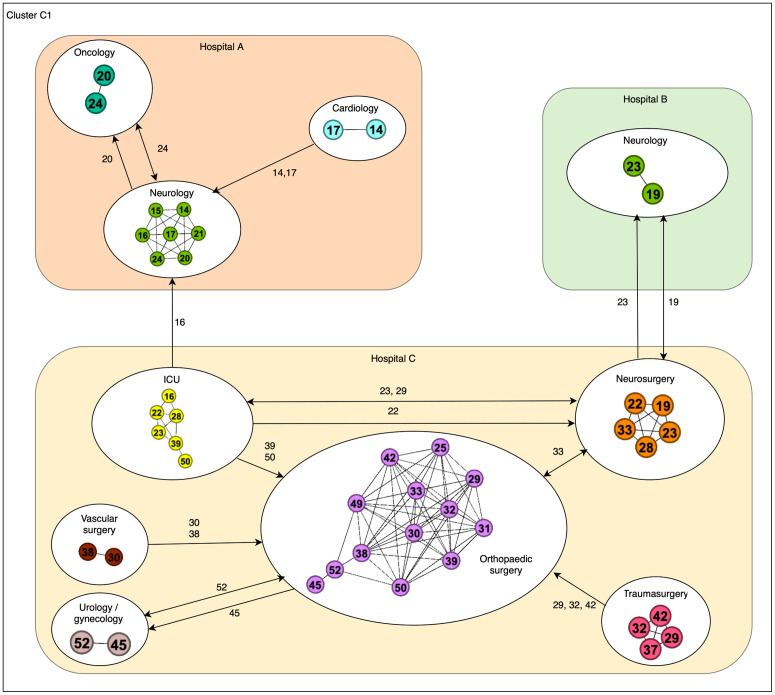
Patients with isolates belonging to cluster C1 and their wards of admission in hospital A, B and C. Only patients with a probable nosocomial transmission link (admission within 1 month of another VREfm positive patient) are shown. Arrows indicate patient transfers. When arrows are bidirectional, this indicates that the patient was transferred in both directions over time.

**Fig 4:**
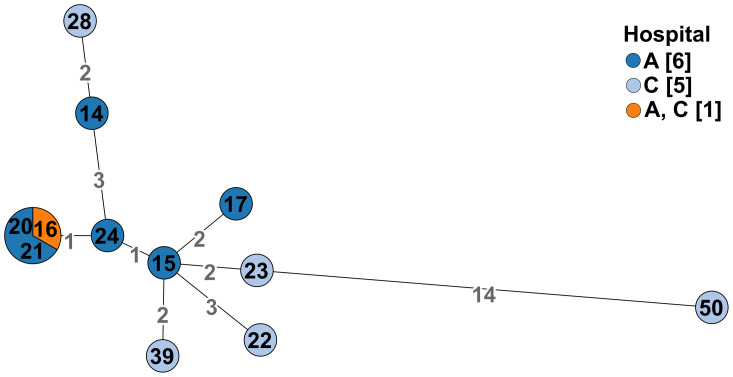
Minimum spanning tree (MST) with allelic distances of VREfm isolates of patients admitted to the same wards as patient 16 in hospital A and C.

The isolates belonging to cluster C1 were detected in 26 patients admitted to three different hospitals: six patients were admitted to Hospital A, 16 to Hospital C, and four were admitted to two different hospitals (three to Hospital B and C; and one to Hospital A and C) (Fig 2, S3 Table). Epidemiological analysis based on previously defined definitions (Table 2) revealed probable intrahospital transmission events involving 19 patients across six wards in hospital C: intensive care (IC), orthopaedic surgery, trauma surgery, urology/gynaecology, vascular surgery, and neurosurgery with an intricate transmission network between these different wards (Fig 3). Patients with isolates belonging to cluster C1 admitted to hospital A (n = 7) and hospital B (n = 2), were all admitted to the neurology ward of their respective hospital within one month of another cluster C1 VREfm positive patient, indicating probable intrahospital transmission events in both hospitals A and B (Fig 3; S3 Table). For one isolate belonging to cluster C1, no epidemiological link could be demonstrated. Additionally, probable interhospital transmission was identified in three out of four patients who were admitted to two different hospitals: These three patients were admitted to the neurosurgery or IC ward of hospital C and the neurology ward of either hospital A or B. (Figs 2 and 3; S3 Table). The first positive VREfm culture of those patients was taken in hospital A (VREfm isolate 16) and hospital B (VREfm isolate 19 and 23). Allelic distances between isolates in cluster C1 from patients admitted to the same wards as patient 16 in hospital A and B had a median of 2 and a range of 0–14 (0–3 when leaving out isolate 50) (Fig 4). Cluster C1 isolates were detected from February 2, 2022 to November 3, 2022 (Fig 2). During this period, VREfm isolates not belonging to cluster C1 were also detected in six other patients admitted to hospital C. These isolates formed a separate cluster, Cluster C2. (Figs 1 and 2; S3 Table). All patients with isolates belonging to cluster C2 were admitted to the geriatric ward in hospital C within one month of each other, pointing to probable intrahospital transmission. None of these patients were admitted to other hospitals (Fig 2; S3 Table). Cluster C3 involved isolates belonging to six patients, all of whom were admitted to hospital D. None of these patients were admitted to other hospitals (Figs 1 and 2; S3 Table). A probable intrahospital transmission event of a clonal VREfm isolate with at least one other patient was identified in all six of the patients of cluster C3.

## Discussion

The present study demonstrates that patient transfers within collaborative healthcare networks can give rise to VREfm transmission networks spanning multiple hospitals. By integrating cgMLST data with epidemiological information in a centralised framework, we were able to delineate transmission clusters, thereby distinguish concurrent outbreaks (e.g., C1 vs C2) and reconstruct interhospital transmission pathways.

As whole genome sequencing is increasingly used to support infection prevention and control, its role is more and more established at the level of the individual hospital [12,13,19–21]. The results of the present study show that the transfer of patients between hospitals can lead to interhospital transmission of VREfm, which is in line with other studies showing that collaborative healthcare networks with patient transfers are a risk factor for MDRO transmission and outbreaks within those networks [22]. Extensive VRE transmission can occur before being noticed due to VRE’s propensity to spread and colonise asymptomatically and its ability to persist op surfaces for extended periods of time (ranging from days to years) [23]. This may complicate the identification of epidemiological relatedness of VRE carriers, potentially delaying outbreak detection, particularly in cases of interhospital transmission.

Without sequence data every newly detected VREfm-carrying patient can be regarded as an isolated case or as part of ongoing transmission, depending on the spatio-temporal relationship with other VREfm-positive patients. Additionally, without a centralised approach, the detection of interhospital spread within collaborative healthcare networks is dependent on the communication between infection control teams of the respective hospitals which is often event-driven (e.g., around patient transfers), retrospective and/or fragmented. Furthermore, even if typing data is available in every involved hospital, it should be standardised in order to be correlated between the hospitals within the network.

While previous studies have identified genomic clusters using WGS-based analyses spanning multiple hospitals, these were based on datasets where epidemiological information was limited to individual institutions, restricting the ability to directly demonstrate interhospital transmission [12,20]. Consequently, there remains a paucity of studies that fully integrate genomic and epidemiological data at the level of a healthcare network. In contrast, our study combines cgMLST data with an epidemiological dataset across multiple hospitals within a single network, allowing genomic clusters to be directly linked to patient movement and thereby supporting interhospital transmission with both genetic and epidemiological evidence. Using this approach, we were able to delineate outbreaks (e.g., the discrimination of the C1 from the C2 cluster) and revealed interhospital transmission events. Due to the centralised nature of the data, it was possible to create a clear overview of the transmission network within and between the involved hospitals.

Our approach could provide infection prevention and control teams with valuable information how and where transmission occurs and support the (de)escalation of infection control measures within hospitals but also between hospitals (e.g., (de)intensification of communication and changes in transfer policies).

Implementation of such approaches requires a centralised sequencing pipeline, data-sharing agreements between involved locations and sufficient infrastructure to ensure timely turnaround time from patient sampling to reports for infection prevention and control teams.

Our results therefore contribute to the growing body of evidence for the value of routine centralised cgMLST-based analysis of WGS data.

A key consideration in interpreting our findings is that common VREfm sequence types such as ST117 and ST80 are widely circulating in healthcare settings and, in themselves, do not imply recent transmission. Previous studies have shown that unrelated isolates within the same ST can differ by hundreds of alleles in cgMLST analyses, underscoring the need for high-resolution typing [24]. In contrast, the isolates in our study showed very limited allelic variation within clusters (typically 1–3 alleles), which—when combined with consistent epidemiological links and the low VREfm background in our country—strongly supports recent transmission rather than coincidental co-occurrence of endemic lineages. While cgMLST clustering thresholds are not universally fixed and may vary between studies, most isolates in our dataset grouped within ≤5 allelic differences. A small number of isolates with larger genetic distances (10–13 alleles) were nevertheless supported by clear epidemiological links, whereas one isolate within 5 alleles lacked such a link. This, in combination with our mean allelic distances within clusters of 1 and within non-clustering isolates of 376 illustrates that our clustering threshold was adequate and reducing the threshold would not alter our conclusions. The absence of a genetic substructure between hospitals, including the patients transferring hospitals, also support the presence of a single transmission network spanning multiple institutions. While there are molecular tools that could leverage WGS data to higher resolution (e.g., split k-mer analysis, SNP-analysis [25]), it is in our case unlikely that this would alter our findings.

The current study has several limitations. Firstly, the relatively low number of VREfm-positive patients detected during the study period outside the context of outbreaks and/or contact tracings introduced selection bias to our results that 86.4% of VREfm isolated from admitted patients were part of a genetic cluster. However, the finding that most VREfm isolates within hospitals are genetically related and indicate within-hospital transmission is in line with studies where clinical isolates were reported separately to reduce selection bias by contact investigations [12,26,27]. An additional limitation is that the discrimination between intrahospital and interhospital transmission can be difficult based on the used criteria. For instance, when the epidemiological analysis of patients with clustering isolates reveals an epidemiological link between the same patients in two or more hospitals (e.g., isolates 19 and 23) it will be difficult to determine in which hospital the transmission took place and therefore whether it should be classified as intrahospital transmission in hospital B or C or as interhospital transmission between both hospitals. However, in the interhospital transmission described between patients in hospital A and C with isolates belonging cluster C1 the possibility of intrahospital transmission could be ruled out. Another limitation is that data on antimicrobial exposure and immunosuppression were not available for this study but may further refine understanding of patient-level risk factors for VREfm acquisition in future studies. Lastly, potential sources and explicit transmission routes were not systematically evaluated in this study. As such, precise reconstruction of transmission chains was not possible. The aim of this study was however not to resolve individual transmission events but to evaluate WGS at the level of a collaborative healthcare network rather than at the level of detailed outbreak reconstruction.

Further studies should evaluate cost-effectiveness and whether our findings apply to other multidrug-resistant organisms.

## Conclusions

Collaborative healthcare networks go hand in hand with intricate transmission networks of VREfm and plead for regional infection prevention and control strategies. Routine, centralised cgMLST-based analysis of WGS data within those networks can play an important role as part of those regional strategies for the detection, tracing and delineation of transmission of VREfm.

## Supporting information

S1 Table Trimming parameters for CLC genomic workbench. (PDF)

S2 Table Assembly parameters for CLC genomic workbench. (PDF)

S3 Table Metadata of all VRE positive patients. (PDF)

S4 Table ST-type versus cluster type distribution of VRE isolates. (PDF)
