# Supplementary material for: Regional VRE surveillance using routine centralised, multicentre whole genome sequencing
Source: PLoS One. 2026 Jun 25;21(6):e0334734. doi: 10.1371/journal.pone.0334734 (PMC13298964; doi:10.1371/journal.pone.0334734)
Supplement: S1 Table — (PDF) [file pone.0334734.s001.pdf]

S1 Table: Trimming parameters for CLC genomic workbench

Trim Sequences (Original name: Trim Reads)	
Trim using quality scores	true
Quality limit	0,01
Trim ambiguous nucleotides	true
Maximum number of ambiguities	2
Automatic read-through adapter trimming	true
Trim adapter list	
Trim homopolymers from 5'	false
Trim homopolymers from 3'	false
polyA	false
polyC	false
polyG	true
polyT	false
Remove 5' terminal nucleotides	false
Number of 5' terminal nucleotides	1
Remove 3' terminal nucleotides	false
Number of 3' terminal nucleotides	1
Remove on first read	true
Remove on second read (for paired reads)	true
Trim to a fixed length	false
Maximum length	150
Trim end	Trim from 3'-end
Discard short reads	false
Minimum length	15
Discard long reads	false
Maximum length	1000
