# Supplementary material for: Regional VRE surveillance using routine centralised, multicentre whole genome sequencing
Source: PLoS One. 2026 Jun 25;21(6):e0334734. doi: 10.1371/journal.pone.0334734 (PMC13298964; doi:10.1371/journal.pone.0334734)
Supplement: S2 Table — (PDF) [file pone.0334734.s002.pdf]

S2 Table: Assembly parameters for CLC genomic workbench

De Novo Assembly	
Mapping mode	Map reads back to contigs (slow)
Update contigs	false
Mismatch cost	2
Insertion cost	3
Deletion cost	3
Length fraction	0,8
Similarity fraction	0,8
Alignment mode	local
Match mode	random
Create list of un-mapped reads	false
Automatic bubble size	true
Bubble size	50
Automatic word size	false
Word size	29
Minimum contig length	500
Guidance only reads	
Perform scaffolding	true
Auto-detect paired distances	true
Create report	true
