# Supplementary material for: Regional VRE surveillance using routine centralised, multicentre whole genome sequencing
Source: PLoS One. 2026 Jun 25;21(6):e0334734. doi: 10.1371/journal.pone.0334734 (PMC13298964; doi:10.1371/journal.pone.0334734)
Supplement: S3 Table — (PDF) [file pone.0334734.s003.pdf]

S3 Table: Metadata of all VRE positive patients

NAdm: Not Admitted, NAss: None Assigned, RP: Rectal/Perineal swab

Patient ID	Collection Date	Time between admission and first culture (days)	Sample Site	Infected	ST_type	Complex type	Cluster type	VanA/B	Total admission days	Hospitals of admission	Wards_of_admission
1	26-01-2022	0	RP	No	80	NAss	NAss	VanA	12	B	Pneumology (Hospital B)
2	17-02-2022	0	RP, Urine	No	80	NAss	NAss	VanA	3	B	Oncology (Hospital B)
3	23-02-2022	NAdm	RP	No	80	NAss	NAss	VanA	N/A	N/A	N/A
4	02-04-2022	2	RP	No	80	NAss	NAss	VanA	11	D	Internal Medicine (Hospital D), Surgery (Hospital D)
5	06-04-2022	NAdm	RP	No	612	2542	NAss	VanA	N/A	N/A	N/A
6	28-05-2022	0	RP	No	117	2505	1	VanA	8	C	Oncology (Hospital C), Internal Medicine (Hospital C)
7	06-06-2022	0	RP	No	18	1898	NAss	VanA	1	C	Vascular Surgery (Hospital C), Gastroenterology (Hospital C)
8	21-06-2022	NAdm	RP	No	117	3640	NAss	VanB	N/A	N/A	N/A
9	29-06-2022	NAdm	RP	No	80	1921	NAss	VanA	N/A	N/A	N/A
10	05-07-2022	0	RP	No	117	NAss	NAss	VanB	N/A	B	Internal Medicine (Hospital B)
11	23-07-2022	0	RP	No	78	6309	NAss	VanA	2	C	Traumasurgery (Hospital C)
12	16-08-2022	0	RP	No	117	71	NAss	VanB	9	D	General Surgical Ward (Hospital D), Internal Medicine (Hospital D), Cardiology (Hospital D)

13	03-09-2022	NAdm	RP, Urine	No	80	NAss	NAss	VanB	N/A	N/A	N/A
14	14-10-2022	5	RP	No	117	2505	1	VanA	21	A	Cardiology (Hospital A), Stroke Unit (Hospital A), Neurology (Hospital A)
15	14-10-2022	1	RP	No	117	2505	1	VanA	10	A	Stroke Unit (Hospital A), Neurology (Hospital A)
16	14-10-2022	11	RP	No	117	2505	1	VanA	21	A, C	Stroke Unit (Hospital A), Neurology (Hospital A), ICU (Hospital C)
17	27-10-2022	5	RP	No	117	2505	1	VanA	26	A	Cardiology (Hospital A), Stroke Unit (Hospital A), Neurology (Hospital A), Traumasurgery (Hospital A)
18	31-10-2022	NAdm	RP	No	NAss	NAss	NAss	VanA	N/A	N/A	N/A
19	02-11-2022	0	RP	No	117	2505	1	VanA	25	B, C	Neurology (Hospital B), Neurosurgery (Hospital C)
20	07-11-2022	Cultured after discharge	RP	No	117	2505	1	VanA	10	A	Stroke Unit (Hospital A), Neurology (Hospital A), Oncology (Hospital A)
21	09-11-2022	Cultured after discharge	RP	No	117	2505	1	VanA	5	A	Stroke Unit (Hospital A), Neurology (Hospital A)
22	12-11-2022	Cultured after discharge	RP	No	117	2505	1	VanA	57	B, C	Cardiology (Hospital B), Cardiology/Cardiothoracic Surgery (Hospital B), ICU (Hospital C), Neurosurgery (Hospital C)
23	17-11-2022	0	RP	No	117	2505	1	VanA	23	B, C	Neurology (Hospital B), Neurosurgery (Hospital C), ICU (Hospital C)
24	20-11-2022	1	RP	No	117	2505	1	VanA	28	A	Pneumology (Hospital A), Stroke Unit (Hospital A), Neurology (Hospital A), Oncology (Hospital A)
25	21-11-2022	0	RP	No	117	2505	1	VanA	22	C	Gastroenterology (Hospital C), Orthopaedic Surgery (Hospital C)
26	23-11-2022	4	RP	No	80	NAss	2	VanA	7	C	Pneumology (Hospital C), Geriatric Ward (Hospital C), Internal Medicine (Hospital C)

27	23-11-2022	4	RP	No	612	NAss	NAss	VanA	8	C	Geriatric Ward (Hospital C)
28	24-11-2022	Cultured after discharge	RP	No	117	2505	1	VanA	38	C	Neurosurgery (Hospital C), ICU (Hospital C), Neurology (Hospital C), GI Surgery (Hospital C)
29	29-11-2022	10	RP	No	117	2505	1	VanA	36	C	URO/GYN (Hospital C), Geriatric Ward (Hospital C), Traumasurgery (Hospital C), Orthopaedic Surgery (Hospital C)
30	29-11-2022	5	RP	No	117	929	1	VanA	28	C	Vascular Surgery (Hospital C), Orthopaedic Surgery (Hospital C)
31	29-11-2022	4	RP	No	117	2505	1	VanA	13	C	Orthopaedic Surgery (Hospital C)
32	29-11-2022	5	RP, Blood	Yes	117	2505	1	VanA	49	C	Orthopaedic Surgery (Hospital C), Pneumology (Hospital C), Geriatric Ward (Hospital C), Cardiology (Hospital C), Coronary Care Unit (Hospital C), Traumasurgery (Hospital C)
33	29-11-2022	25	RP	No	117	2505	1	VanA	68	C	Orthopaedic Surgery (Hospital C), Neurosurgery (Hospital C)
34	30-11-2022	6	RP	No	80	NAss	2	VanA	43	C	Internal Medicine (Hospital C), Gastroenterology (Hospital C), Cardiology (Hospital C), Orthopaedic Surgery (Hospital C), Pneumology (Hospital C), Geriatric Ward (Hospital C)
35	30-11-2022	13	RP	No	80	NAss	2	VanA	42	C	Cardiology (Hospital C), Geriatric Ward (Hospital C)
36	30-11-2022	2	RP	No	80	NAss	2	VanA	13	C	Geriatric Ward (Hospital C)
37	30-11-2022	Cultured after discharge	RP	No	117	2505	1	VanA	92	C	Traumasurgery (Hospital C)
38	07-12-2022	3	RP	No	117	2505	1	VanA	25	C	Vascular Surgery (Hospital C), Orthopaedic Surgery (Hospital C)
39	07-12-2022	12	RP	No	117	2505	1	VanA	31	C	ICU (Hospital C), Orthopaedic Surgery (Hospital C)
40	11-12-2022	13	RP	No	80	NAss	2	VanA	15	C	Geriatric Ward (Hospital C)

41	12-12-2022	6	RP	No	NAss	7088	3	VanA	9	D	Internal Medicine (Hospital D), Surgery (Hospital D)
42	14-12-2022	1	RP	No	117	2505	1	VanA	7	C	Traumasurgery (Hospital C), Orthopaedic Surgery (Hospital C)
43	21-12-2022	Cultured after discharge	RP	No	80	NAss	2	VanA	5	C	Geriatric Ward (Hospital C)
44	22-12-2022	2	RP	No	NAss	7088	3	VanA	11	D	Internal Medicine (Hospital D), Neurology (Hospital D)
45	28-12-2022	0	Skin, Scrotum	No	117	2505	1	VanA	40	C	Orthopaedic Surgery (Hospital C), URO/GYN (Hospital C)
46	28-12-2022	Cultured after discharge	RP	No	NAss	7088	3	VanA	32	D	Surgery (Hospital D), Internal Medicine (Hospital D)
47	02-01-2023	6	RP	No	NAss	7088	3	VanA	21	D	Internal Medicine (Hospital D), Neurology (Hospital D)
48	03-01-2023	Cultured after discharge	RP	No	NAss	7088	3	VanA	24	D	Internal Medicine (Hospital D), Neurology (Hospital D), ICU (Hospital D), Pneumology (Hospital D)
49	05-01-2023	14	RP	No	117	2505	1	VanA	17	C	Orthopaedic Surgery (Hospital C)
50	12-01-2023	19	RP	No	117	2505	1	VanA	30	C	ICU (Hospital C), Orthopaedic Surgery (Hospital C)
51	18-01-2023	Cultured after discharge	RP	No	NAss	7088	3	VanA	11	D	Internal Medicine (Hospital D), Neurology (Hospital D)
52	02-02-2023	8	RP	No	117	2505	1	VanA	19	C	Orthopaedic Surgery (Hospital C), URO/GYN (Hospital C), GI Surgery (Hospital C)
53	28-02-2023	NAdm	Feces	No	117	NAss	NAss	VanB	N/A	N/A	N/A
54	15-03-2023	Cultured after discharge	RP	No	736	NAss	NAss	VanA	N/A	B	Cardiology/Cardiothoracic Surgery (Hospital B)
55	20-05-2023	0	RP	No	80	6684	NAss	VanA	21	B	Orthopaedic Surgery (Hospital B)

56	05-07-2023	NAdm	RP	No	80	NAss	NAss	VanA	N/A	N/A	N/A
57	06-07-2023	NAdm	RP	No	80	3213	NAss	VanA	N/A	N/A	N/A
