# Supplementary material for: Regional VRE surveillance using routine centralised, multicentre whole genome sequencing
Source: PLoS One. 2026 Jun 25;21(6):e0334734. doi: 10.1371/journal.pone.0334734 (PMC13298964; doi:10.1371/journal.pone.0334734)
Supplement: S4 Table — (PDF) [file pone.0334734.s004.pdf]

S4 Table: ST-type versus cluster type distribution of VRE isolates.

Cluster type / ST-type	1	2	3	None	Total
18				1	1
78				1	1
80		6		9	15
117	26			4	30
612				2	2
736				1	1
Unassigned			6	1	7
Total	26	6	6	19	57
